# Zoonotic Tick‐Borne Pathogens in *Ixodes ricinus* Complex (Acari: Ixodidae) From Urban and Peri‐Urban Areas of Kosovo

**DOI:** 10.1111/zph.13197

**Published:** 2024-12-08

**Authors:** Ina Hoxha, Betim Xhekaj, Genc Halimi, Michiel Wijnveld, Margarida Ruivo, Driton Çaushi, Albana Matoshi, Adelheid G. Obwaller, Bernhard Jäger, Martin Weiler, Julia Walochnik, Kurtesh Sherifi, Edwin Kniha

**Affiliations:** ^1^ Institute of Specific Prophylaxis and Tropical Medicine, Center for Pathophysiology, Infectiology and Immunology Medical University Vienna Vienna Austria; ^2^ Faculty of Agriculture and Veterinary University of Prishtina “Hasan Prishtina” Prishtina Republic of Kosovo; ^3^ Institute for Hygiene and Applied Immunology, Center for Pathophysiology, Infectiology and Immunology Medical University of Vienna Vienna Austria; ^4^ Faculty of Medicine University of Prishtina “Hasan Prishtina” Prishtina Republic of Kosovo; ^5^ Division of Science, Research and Development Federal Ministry of Defence Vienna Austria; ^6^ CBRN Defence Centre Austrian Armed Forces Korneuburg Austria

**Keywords:** *Anaplasma*, *Babesia*, *Borrelia*, public health, reverse line blotting, *Rickettsia*

## Abstract

**Introduction:**

*Ixodes ricinus* , the castor bean tick, is the most prevalent tick species in Europe. It favours habitats such as shrubs, deciduous and mixed forests, but can also be found in urban environments. Due to its high vector competence, it is of enormous veterinary as well as medical importance, transmitting tick‐borne encephalitis (TBE) virus, *Borrelia burgdorferi* s. l., the causative agent of lyme borreliosis, *Rickettsia* spp. and *Anaplasma phagocytophilum* amongst many other pathogens. In Kosovo, *I. ricinus* is the predominant species and a few studies, mostly based on human and animal seroprevalences, indicate the circulation of tick‐borne pathogens. However, data on pathogens in *I. ricinus* are scarce in Kosovo, particularly in urban settings. This study aimed to provide first insights into the circulation of tic‐kborne pathogens in *I. ricinus* from urban and peri‐urban areas in Kosovo.

**Methods:**

Urban and peri‐urban areas were sampled by flagging 150 m transects. In total, 197 ticks were morphologicaly identified as *I. ricinus*, and consequently DNA and RNA were isolated. All individuals were screened for the presence of tick‐borne pathogens by using reverse line blotting (RLB) hybridisation.

**Results:**

DNA of nine different pathogens from four genera including *Borrelia* spp., *Rickettsia* spp., *Anaplasma* spp. and *Babesia* spp. was detected in 60 (33.5%) specimens. The most frequently detected pathogens were *Rickettsia* spp. (16.2%), followed by *Borrelia* spp. (11.7%). Altogether, 54 single infections, 11 double infections and 1 triple infection were observed.

**Conclusions:**

We provide first data on genotyping of *B. burgdorferi* sensu lato as well as the detection of *Anaplasma*, *Babesia* and *Rickettsia* from *I. ricinus* in this country. The data underline that particularly recreational (peri‐)urban areas could facilitate the spillover of zoonotic tick‐borne pathogens to humans in Kosovo and provide baseline data for future surveys.

**Summary**

- DNA of multiple tick‐borne pathogens of medical and veterinary relevance were detected in *Ixodes ricinus* in Kosovo.
- The findings highlight that particularly (peri‐)urban areas can be a source of infection with tick‐borne pathogens for the local population and domestic animals.
- The data suggests that tick‐borne diseases are clearly underreported in Kosovo, and broader surveys, including vector surveillance and serosurveys, are necessary to assess species and pathogen diversity.

## 1 Introduction

*Ixodes ricinus* (Acari: Ixodidae) is the most widespread tick species in Europe and can transmit viral, bacterial as well as protozoan pathogens of medical and veterinary importance (Rizzoli et al. 2014). Its long‐lasting life cycle involves three questing stages (larvae, nymphs and adults) that attach to a host for blood feeding. *Ixodes ricinus* is reported to be primarily present in areas with shrubs and deciduous and mixed forests that show a high abundance of small to large wild vertebrate hosts. However, stable tick populations have been observed in urban and peri‐urban areas in many European countries (Hansford et al. 2022; Schötta et al. 2023).

*Ixodes ricinus* has a huge host spectrum feeding on mammals, birds and lizards. While rodents are amongst the most important hosts for sub‐adult stages, adults prefer domestic and medium‐sized and large wild animals, including humans. However, when specific hosts are absent, the host spectrum can be shifted towards the most prevalent fauna in a respective area (Gray 1998; Jaenson and Tälleklint 1996; Bowman and Nutall 2008).

Amongst many pathogens transmitted by *I. ricinus* , tick‐borne encephalitis (TBE) virus (Flaviviridae) is the most important viral agent of human infection in Europe and Eastern and Central Asia. While often asymptomatic, TBEV infections can lead to neurologic diseases such as meningitis, encephalitis, encephalomyelitis and radiculitis (Heinz et al. 2013; Kunze 2013). The natural transmission cycles mainly occur in sylvatic habitats involving vector ticks and reservoir hosts, however, shifts from previously natural habitats to urban areas have been reported (Korenberg, Cerný, and Daniel 1984).

Lyme borreliosis (LB), caused by the gram‐negative spirochetes of the *Borrelia burgdorferi* sensu lato (s. l.) complex, is the most abundant tick‐borne disease in the Northern Hemisphere and closely correlates to the vector species' distribution (Durden and Beati 2014). Of currently over 20 *Borrelia* species within the complex, *B. afzelii* , *B. burgdorferi* sensu stricto (s. s.) and *B. garinii* are most often associated with localised, disseminated and chronic manifestations of LB in Europe. Others such as *B. valaisiana* have been detected in samples from single cases of LB and *B. lusitaniae* is of unclear clinical role (Franke, Hildebrandt, and Dorn 2013; Stanek and Reiter 2011; Steinbrink et al. 2022). The infection risk is high in forest ecosystems, but also in recreational areas (e.g., city parks and gardens). Particularly, activities such as hiking, jogging or dog walking can enhance the risk of infection in these habitats (Hubálek 2009).

The gram‐negative, aerobic, obligate intracellular bacteria *Rickettsia* spp. of the spotted fever group (SFG) are veterinary and medically important tick‐borne pathogens. Some species, namely *Rickettsia slovaca* and *Rickettsia raoultii* , are known to cause scalp eschar, facial edema and cervical lymphadenopathy (tick‐borne lymphadenopathy, TIBOLA) or *Dermacentor*‐borne necrosis erythema and lymphadenopathy (DEBONEL) (Parola et al. 2009). While *Rickettsia monacensis* can cause fever, headache, general discomfort, joint pain and erythematous rash, the risk of human infections by *Rickettsia helvetica* is unclear (Jado et al. 2007).

Amongst others, the intracellular bacterium *Anaplasma phagocytophilum* can cause mostly self‐limiting infections, human granulocytic anaplasmosis (HGA), however, severe and even fatal cases of infections are known from immunocompromised patients (Dumler, Barat, et al. 2007; Dumler, Madigan, et al. 2007).

The zoonotic protozoan parasites of the genus *Babesia* (Apicomplexa) are the causative agents of babesiosis. Symptoms comprise flu‐like to malaria‐like illness including malaise, chills, myalgia, anaemia, fatigue and fever. Particularly splenectomised individuals can experience life‐threatening complications (Homer et al. 2000).

In Kosovo, various tick species that can transmit pathogens of veterinary, and medical importance have been reported. In a study from 2014 and 2015, the most prevalent species of questing ticks by flagging was *I. ricinus* , followed by *Dermacentor marginatus* and *Haemaphysalis* spp. However, only 1.5% of collected *I. ricinus* ticks were positive for *B. burgdorferi* s. l. DNA and all were negative for TBEV RNA (Sherifi et al. 2018). A recent study on selected vector‐borne pathogens revealed an *Anaplasma* spp. seroprevalence of around 25% in dogs, whereas *Ehrlichia* spp. (0.7%) and *B. burgdorferi* s. l. (1.3%) seroprevalence was rather low (Sinani et al. 2020). First data on human LB in Kosovo in 2021 indicated the circulation of *B. burgdorferi* s. l. in the human population (Ponosheci‐Biçaku et al. 2021).

Despite the high veterinary and medical relevance of tick‐borne diseases, data are scarce in Kosovo, particularly in urban and peri‐urban settings, especially in most human frequented recreational parks. Therefore, we aimed to provide first data on tick‐borne pathogens in *I. ricinus* from urban and peri‐urban areas in Kosovo.

## 2 Material and Methods

### 2.1 Study Sites

**FIGURE 1:**
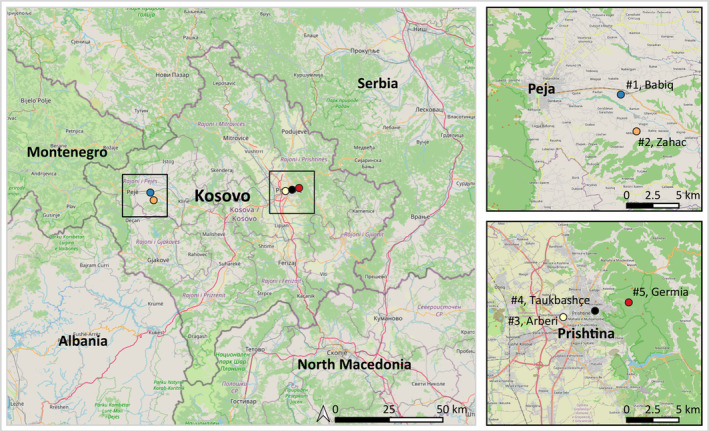
Tick collection sites in Kosovo (left). Magnification of collection sites (#1–#5) are shown in black frames (right). The map was created with QGIS (QGIS Development Team 2019). The map of Kosovo is taken from Open Street Map implemented in QGIS.

The study was carried out at five different urban or peri‐urban locations in April 2022 in Kosovo (Figure 1).

Location #1 (Babiq) (42.622, 20.406) can be described as a peri‐urban area in the eastern part of Kosovo, 10 km southeast of Peja, the largest city in the respective district. An approx. 200 × 200 m area encircled by deciduous trees next to an animal farm housing sheep, cattle, dogs, cats and chicken was sampled.

Location #2 (Zahac) (42.654, 20.387) is located in a peri‐urban area in eastern Kosovo 5 km east of Peja. A 100 × 100 m area encircled by deciduous trees next to animal farms and private households were sampled. Dogs, cats, goats and chicken were present at local farms in the surrounding areas.

Location #3 (Arberi) (42.660, 21.143) exhibits an urban, undeveloped area between newly built apartment houses in the city of Prishtina, the capital of Kosovo. The area (150 × 50 m) consisted of wild weed, bushes and litter. Stray dogs were frequently observed and children playing close‐by.

Location #4 (Taukbashçe) (42.665, 21.180) represents a public recreational park in the city of Prishtina, which is used by locals for recreation, sports as well as dog‐walking. The park displays an urban area of approx. 500 × 100 m with many deciduous trees and a small forest on the southern border of the park. While the park is regularly sprayed with insecticides in the summertime, the bordering forest area is kept naturally and particularly used for dog walking.

Location #5 (Germia) (42.672, 21.219) is a recreational peri‐urban area (Germia National Park, ~23 km^2^) at the northeastern outskirts of Prishtina. The mixed forest of the national park is used by local residents for hiking and dog walking. An area (150 × 50 m) close to a parking lot of a popular restaurant was sampled.

### 2.2 Tick Collection and Morphological Identification

Questing ticks were sampled by flagging with white flannel (1 × 1 m) along an approx. 150 m transect at single occasions at every location. Cloth examination was performed at 10‐m intervals to avoid tick detachment. Collected ticks were put in 8 mL tubes with screw caps, transferred in dry ice and stored at −80°C until further inspection. Ticks were morphologically identified using a stereomicroscope and the identification keys by Estrada‐Peña, Mihalca, and Petney (2018).

### 2.3 Nucleic Acid Extraction and Molecular Identification

Prior to nucleic acid isolation, ticks were washed in 70% ethanol and cleaned with fine brushes, if necessary. For RNA and DNA extraction, ticks were longitudinally cut into two halves. DNA was isolated from individual halves of ticks using a Qiagen DNeasy Blood and Tissue Kit (Qiagen, Hilden, Germany) by incubating in 180 μL ATL buffer and 20 μL Proteinase K overnight following the manufacturer's instructions.

For RNA isolation, halves of ticks were pooled (maximum of five individuals) by sex and location, 180 μL phosphate buffer saline (PBS) was added and crushed with 3 mm stainless steel beads for 5 min at 6000×*g*. Thereafter, the homogenate was centrifuged at 18,000×*g* for 5 min and the supernatant was transferred to a new tube. Consecutively, a Qiagen RNeasy Mini Kit (Qiagen, Hilden, Germany) was used, adding 560 μL AVL‐AVE Lysis buffer (containing RNA‐carrier) to the homogenate, followed by 350 μL of 70% Ethanol, only mixing by pipetting, all following steps were done based on the manufacturer's instructions. The final elution volume was 50 μL and the RNA eluate was stored at −20°C until further use.

Molecular identification of chosen ticks was based on amplification of a 16S rRNA gene segment using the primer combination 16S+1 (5′‐CTGCTCAATGATTTTTTAAATTGCTGTGG‐3′) and 16S−1 (5′‐CCGGTCTGAACTCAGATCAAGT‐3′) published by Black and Piesman (1994). The PCR conditions were 94°C for 5 min initial denaturation followed by 38 cycles of 94°C for 1 min, 52°C for 1 min, and 72°C for 1 min and a final elongation at 72°C for 10 min. For PCR amplifications a 2x EmeraldAmp GT PCR Master Mix (Takara Bio Europa SAS, Saint‐Germain‐en‐Laye, France) with 2 μL template DNA and sterile H_2_O adding up to a final volume of 25 μL was used.

All PCR amplifications were run on an Eppendorf Mastercycler (Eppendorf AG, Hamburg, Germany). Bands were analysed with a Gel DocTM XR+ Imager (Bio‐Rad Laboratories Inc., California, U.S.A.), cut out, purified with an IllustraTM GFXTM PCR DNA and Gel Purification Kit (GE Healthcare, Buckinghamshire, UK) and sent to Microsynth Austria GmbH for Sanger sequencing.

Sequences were obtained from both strands, aligned with Clustal X 2.1 (Larkin et al. 2007), and a consensus sequence was generated in GenDoc 2.7.0 (Nicholas 1997). The obtained sequences were uploaded to GenBank (PP231780–PP231807) and compared to available sequences in the GenBank database using the Basic Local Alignment Search Tool (BLAST) (https://blast.ncbi.nlm.nih.gov/Blast.cgi).

### 2.4 Pathogen Screening

#### 2.4.1 Reverse Line Blotting (RLB)

The sampled ticks were screened for the presence of the following pathogens: *B. burgdorferi* s. l., *Anaplasma*/*Ehrlichia* spp., *Babesia*/*Theileria* spp., *Rickettsia* spp., using the PCR‐RLB hybridization method. Five genus‐specific RLB‐PCRs were carried out as reported previously (Schötta et al. 2017; Wijnveld et al. 2016), using the biotinylated primer pairs shown in Table 1. Each PCR reaction mix with a 25 μL total volume, contained: 5 μL (5×) of Phire reaction buffer, 200 nmol/L of each dNTP (Solis BioDyne, Tartu, Estonia), 400 nmol/L of each primer per specific primer pair, 0.125 μL Phire Hot Start II DNA Polymerase (Thermo Scientific, Vienna, Austria), PCR‐grade water (Sigma‐Aldrich, Vienna, Austria) and lastly 2.5 μL of template DNA (Wijnveld et al. 2021). The resulting amplicons were analysed using RLB hybridisation as described previously (Gubbels et al. 1999).

**TABLE 1:** List of primers used for the reverse line blot.

Pathogens	(5′–3′) Sequence	Targeted region	References
*Anaplasma/Ehrlichia* spp.	GGAATTCAGAGTTGGATCMTGGYTCAG (Biotin‐) CGGGATCCCGAGTTTGCCGGGACTTYTTCT	16S rRNA gene	Bekker et al. (2002) and Schouls et al. (1999)
*Babesia*/*Theileria* spp.	GACACAGGGAGGTAGTGACAAG (Biotin‐)CTAAGAATTTCACCTCTGACAGT	18S rRNA gene	Georges et al. (2001)
*Borrelia burg‐dorferi s. l*.	ACCATAGACTCTTATTACTTTGACCA (Biotin‐)GAGAGTAGGTTATTGCCAGGG	5S‐23S rRNA intergenic spacer	Rijpkema et al. (1995)
*Rickettsia* spp.	GAACGCTATCGGTATGCTTAACACA (Biotin‐)CATCACTCACTCGGTATTGCTGGA	16S rRNA gene	Christova et al. (2003) and Nijhof et al. (2007)
	GATAGGTCRGRTGTGGAAGCAC (Biotin‐)TCGGGAYGGGATCGTGTGTTTC	23S‐5S rRNA intergenic spacer	Jado et al. (2006)

#### 2.4.2 *Francisella tularensis* Detection by PCR

For the detection of *F. tularensis* DNA, the primers TUL4‐435 (5′‐GCTGTATCATCATTTAATAAACTGCTG‐3′) and TUL4‐863 (5′‐TTGGGAAGCTTGTATCATGGCACT‐3′) published by Sjöstedt et al. (1997) were used with following PCR conditions: 94°C for 5 min, followed by 40 cycles of 94°C for 1 min, 54°C for 1 min and 72°C for 1 min, and a final elongation of 10 min at 72°C was applied. Five μL of template DNA were used for all samples. Sterile H_2_O was used as a negative control and *F. tularensis* DNA was used as a positive control.

#### 2.4.3 Pan‐Flavivirus Screening by qPCR

To test for the presence of TBEV RNA, a reverse transcriptase (RT) qPCR with a published ‘universal’ flavivirus primer set PF1S (5′‐TGYRTBTAYAACATGATGGG‐3′) and PF2Rbis (5′‐GTGTCCCADCCDGCDGTRTC‐3′) (Moureau et al. 2007) using SYBR green and a Luna Universal One‐Step RT‐qPCR Kit (New England Biolabs, Ipswich, MA, USA) with the following PCR conditions: 55°C for 10 min, followed by 45 cycles of 95°C for 1 min, 95°C for 10 s and 50°C for 1 min. For all samples 2.5 μL of RNA template was used. Sterile H_2_O was used as a negative control and RNA extracted from a live Yellow fever vaccine (Stamaril) was used as a positive control.

### 2.5 Statistical Analysis

All data were analysed using Microsoft Excel 16.82 for Mac and R 3.6.2 (R Core Team 2023). To compare infection rates of ticks (overall and by pathogen) between locations, we applied a Fisher's exact test. A two‐sided *p*‐value < 0.05 was considered statistically significant.

## 3 Results

### 3.1 Tick Species

Altogether, 197 specimens were caught, of which 32 (16.2%) were nymphs and 165 (83.8%) were adults. The latter comprised 79 (40.1%) females and 86 (43.7%) males. The number of caught specimens varied between trapping sites, being highest at location #4 (72) and lowest at location #1 (9) (Table 2).

**TABLE 2:** Number of collected ticks by sex, developmental stage and sampling site.

Sampling site	Nymph (*n* = 32)	Female (*n* = 79)	Male (*n* = 86)
#1, Babiq (*n* = 9)	2 (6.3%)	4 (5.1%)	3 (3.5%)
#2, Zahac (*n* = 22)	6 (18.8%)	7 (8.9%)	9 (10.5%)
#3, Arberi (*n* = 28)	0	11 (13.9%)	17 (19.8%)
#4, Taukbashçe (*n* = 72)	2 (6.3%)	40 (50.6%)	30 (34.9%)
#5, Germia (*n* = 66)	22 (68.8%)	17 (21.5%)	27 (31.4%)

All individuals were morphologically identified as *I. ricinus* complex. Of these, 28 specimens were barcoded based on 16S rDNA sequences. Sequence identities compared to reference sequences of *I. ricinus* ranged from 99.51% to 100%.

### 3.2 Pathogen Screening

Of 197 specimens, 66 (33.5%) were positive for at least one pathogen, comprising 10 (10/32; 31.3%) positive nymphs, 25 (25/79; 31.6%) positive females and 31 (31/86; 36.1%) positive males. Altogether, nine different pathogens from four genera were detected. The positivity rate was 16.2% (32/197) for *Rickettsia* spp., 11.7% (23/197) for *B. burgdorferi* s. l., 7.1% (14/197) for *A. phagocytophilum* , and 5.1% (10/197) for *Theileria* (*Babesia*) *microti*. Reverse line blotting identified four *Rickettsia* species, namely *R. helvetica* , *R. monacensis* , *R. raoultii* and *R. slovaca* as well as three *Borrelia* species, namely *B. afzelii* , *B. lusitaniae* and *B. valaisiana* . No specimens were positive for *F. tularensis* DNA or Flavivirus RNA.

### 3.3 Co‐Infections

Of all positive specimens, 54 single infections, 11 double infections, and 1 triple infection were observed (Table 3). While double infections were detected in nymphs, females and males, the triple infection was only observed in one female specimen, being infected with *B. afzelii* , *B. lusitaniae* and *R. helvetica* .

**TABLE 3:** Single pathogens and co‐infections detected in the collected ticks.

Pathogen	Nymphs (*n* = 32)^a^	Female (*n* = 79)^a^	Male (*n* = 86)^a^	Total (*n* = 197)^b^
Single infection				
*A. phagocytophilum*	2 (6.3%)	5 (6.3%)	7 (8.1%)	14 (7.1%)
*Theileria* (*B*.) *microti*	2 (6.3%)	4 (5.1%)	4 (4.7%)	10 (5.1%)
*Borrelia* spp.	3 (9.4%)	9 (11.4%)	11 (12.8%)	23 (11.7%)
*B. afzelii*	—	5 (6.3%)	8 (9.3%)	13 (6.6%)
*B. lusitaniae*	3 (9.4%)	3 (3.8%)	3 (3.5%)	9 (4.6%)
*B. valaisiana*	—	1 (1.3%)	—	1 (0.5%)
*Rickettsia* spp.	5 (15.6%)	11 (13.9%)	16 (18.6%)	32 (16.2%)
*R. helvetica*	2 (6.3%)	3 (3.8%)	9 (10.5%)	14 (7.1%)
*R. monacensis*	1 (3.1%)	4 (5.1%)	5 (5.8%)	10 (5.1%)
*R. raoultii*	2 (6.3%)	4 (5.1%)	1 (1.2%)	7 (3.6%)
*R. slovaca*	—	—	1 (1.2%)	1 (0.5%)
Double infection				
*A. phagocytophilum* + *R. monacensis*	—	—	1 (1.2%)	1 (0.5%)
*A. phagocytophilum* + *R. helvetica*	—	—	1 (1.2%)	1 (0.5%)
*Th. microti* + *B. afzelii*	—	—	2 (2.3%)	2 (1.0%)
*Th. microti* + *R. monacensis*	—	—	1 (1.2%)	1 (0.5%)
*B. afzelii* + *R. helvetica*	—	—	2 (2.3%)	2 (1.0%)
*B. afzelii* + *R. monacensis*	—	2 (2.5%)	—	2 (1.0%)
*B. lusitaniae* + *R. monacensis*	1 (3.1%)	—	—	—
*B. lusitaniae* + *R. raoultii*	1 (3.1%)	1 (1.3%)	—	2 (1.0%)
Triple infection				
*B. afzelii* + *B. lusitaniae* + *R. helvetica*	—	1 (1.3%)	—	1 (0.5%)

^a^ No. and % of infected stage.

^b^ Total no. and % infected.

Co‐infections involving *A. phagocytophilum* or *T*. (*B*.) *microti* were only observed in males, whereas co‐infections with *Borrelia* spp. and *Rickettsia* spp. were observed in nymphs, females and males (Table 3).

### 3.4 Detected Pathogens by Location

Prevalence of infected ticks varied by location, being highest (54.5%) at location #2, followed by location #4 (34.7%), location #5 (33.3%), location #1 (22.2%) and being lowest (17.7%) at location #3. *Theileria* (*B*.) *microti* and *B. valaisiana* were only detected in ticks from location #5, Germia, whereas *R. slovaca* was only detected at location #4, Taukbashçe (Table 4). The highest diversity of pathogens was detected at location #5, showing seven different pathogens, while the lowest was observed at location #1 being only positive for *B. lusitaniae* (Table 4).

**TABLE 4:** Detected tick‐borne pathogens by location.

Location (no. of ticks)	*Anaplasma*	*Babesia*	*B. burgdorferi* s. l.			*Rickettsia* spp.				Total
	Aph	B(Th)m	Bafz	Blus	Bval	Rhel	Rmon	Rrao	Rslo	
#1, Babiq (9)^a^	—	—	—	2	—	—	—	—	—	2
#2, Zahac (22)	1	—	1	5	—	5	3	1	—	16
#3, Arberi (28)	11	—	1	2	—	—	1	1	—	16
#4, Taukbashçe (72)	—	—	7	—	—	7	—	2	1	17
#5, Germia (66)	2	10	4	—	1	2	6	3	—	28

Abbreviations: Aph, *A. phagocytophilum* ; B(Th)m, *B. microti* ; Bafz, *B. afzeli*; Blus, *B. lusitaniae* ; Bval, *B. valaisiana* ; R. slo, *R. slovaca* ; Rhel, *R. helvetica* ; Rmon, *R. monacensis* ; Rrao, *R. raoultii* .

^a^ Number of collected ticks.

**FIGURE 2:**
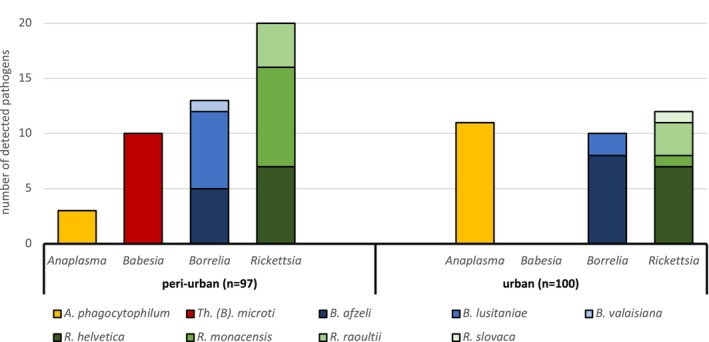
Pathogen diversity in ticks from peri‐urban and urban locations.

Altogether, a higher but not significant infection rate was detected at peri‐urban (#1, #2, #5) compared to urban (#3 and #4) locations (47.4% vs. 33.0%, *p* = 0.06). Also, more double infections (8.3% vs. 3.0%, *p* = 0.13) and the only triple infection were observed at peri‐urban locations. The *A. phagocytophilum* prevalence was significantly higher in ticks from urban locations (3.1% vs. 11.0%, *p* = 0.05). *Theileria* (*B*.) *microti* was only detected at one peri‐urban location (Germia National park). Neither the *Borrelia* spp. (13.4% vs. 10.0%, *p* = 0.5) nor the *Rickettsia* spp. prevalence (20.6% vs. 12.0%, *p* = 0.12) differed significantly between peri‐urban and urban sites (Figure 2).

## 4 Discussion

In this study, we report the detection of DNA of several zoonotic tick‐borne pathogens in *I. ricinus* from Kosovo. To the best of our knowledge, we provide the first data on genotyping of *B. burgdorferi* s. l. as well as the detection of *Anaplasma*, *Babesia* and *Rickettsia* from *I. ricinus* in Kosovo. We also provide the first 16S rDNA sequences of *I. ricinus* from Kosovo for future studies.

To date, only one study showed the presence of *Rickettsia* in ticks from Kosovo, namely Astrakhan fever rickettsia ( *Rickettsia conorii* ) in four *Rhipicephalus sanguineus* specimens (Fournier et al. 2003), but no data on human rickettsiosis is available. We report for the first time DNA of four *Rickettsia* species in *I. ricinus* from Kosovo. Of these, *R. raoultii* and *R. slovaca* are of medical importance by causing TIBOLA, involving an inoculation eschar on the scalp and cervical lymphadenopathies after a tick bite, however, the principal vectors are ticks of the genus *Dermacentor* (Parola et al. 2009), which might be present at the sampled trapping sites, with low to little activity in April and thus not observed. In addition, *R. monacensis* has previously been identified as the causative agent of a Mediterranean spotted fever‐like illness in Italy and Spain (Jado et al. 2007; Madeddu et al. 2012), and the pathogenicity of *R. helvetica* has been suspected, but never proven (Brouqui et al. 2007). The observed low *R. slovaca* and *R. raoultii* prevalences can be attributed to the fact that *I. ricinus* is not the main vector for these *Rickettsia* species, and the presence of *Dermacentor* spp. in Kosovo should be assessed in future studies. However, the detection of *R. monacensis* in ticks from both urban and peri‐urban areas, particularly in nymphs and females, may pose a public health risk for the local human population. Clearly, tick‐borne rickettsiae of the Spotted Fever group (SFGR) are emerging zoonotic pathogens in Europe, including the Balkans. However, data is rather scarce and only a few studies have reported human rickettsiosis from neighbouring countries such as Serbia (Banović et al. 2023). Considering the frequent detection of *Rickettsia* spp. in ticks and the underreported nature of SFGR in the Balkans, on‐going monitoring and surveillance should be established in Kosovo and its neighbouring countries.

While a few previous studies have indicated the presence of *B. burgdorferi* s. l. in Kosovo, we report the presence of three different species of the *B. burgdorferi* complex, namely *B. afzelii* being of highest abundance, followed by *B. lusitaniae* and few *B. valaisiana* . Particularly *B. afzelii* is amongst the most reported *Borrelia* species in Europe and of medical relevance by causing erythema migrans (EM) and acrodermatitis chronica atrophicans (ACA) (Franke, Hildebrandt, and Dorn 2013). Sherifi et al. (2018) reported 1 of 134 (0.8%) *I. ricinus* from Kosovo positive for *B. burgdorferi* s. l., which is much lower compared to the detected mean prevalence of 11.7% of our study. However, a meta‐analysis including 155 European studies by Rauter and Hartung (2005) showed an overall mean *Borrelia* infection rate of 13.6% in *I. ricinus* , which is in line with our study. The observed seroprevalence of 4.3% in dogs from the same regions (Prishtina and Peja) amongst those sampled in our study highlights the circulation of *Borrelia* in dogs, and particularly the high number of stray dogs may facilitate the transmission (Sinani et al. 2020). Our detected *Borrelia* infection rates in peri‐urban and urban areas suggest a risk of infection also in urban areas, for example Taukbashçe (location #4), which is used as a recreational area by the local population, also for dog walking. This is supported by a study from Ponosheci‐Biçaku et al. (2021), who reported 96% of tick bites within their study cohort to be related to recreational activities in Kosovo.

*Anaplasma phagocytophilum* prevalence in questing ticks varies considerably between countries, from 0.7% in Austria (Schötta et al. 2017) or Spain (Remesar et al. 2021) to 3.4% in Romania (Matei et al. 2015) and 13.9% in Serbia (Milutinović et al. 2008). We evaluated an overall prevalence of 7.2%, being significantly higher in urban areas compared to peri‐urban areas. Notably, the majority of infected ticks originated from location #3 (Arberi), which is an undeveloped area between newly built apartment houses in the city of Prishtina. In Europe, roe deer ( *Capreolus capreolus* ) are the suspected main reservoir hosts for *A. phagocytophilum* , but also red deer ( *Cervus elaphus* ), wild boar ( *Sus scrofa* ), red foxes ( *Vulpes vulpes* ), small rodents, hedgehogs ( *Erinaceus europaeus* ) and even birds (e.g., blackbird, *Turdus merula* ) may play a role (Dugat et al. 2015). Similarly to other studies (e.g., Schorn et al. 2011), ungulates are absent at the urban locations of our study, particularly location #3, which suggests the role of other animals as reservoir hosts. High infection rates have been observed in rodents in Switzerland, foxes ( *V. vulpes* ) in Austria and Czech Republic and hedgehogs ( *Erinaceus europaeus* ) in Germany (Liz et al. 2000; Petrovec et al. 2003; Skuballa et al. 2010). Considering the observed presence of stray dogs around Arberi location, also dogs might serve as reservoirs at urban localities, but more data are needed to identify the role of domestic animals in enzootic *A. phagocytophilum* transmission cycles. However, we would like to stress that our analysis cannot discriminate between strains, and thus the pathogenicity of detected *A. phagocytophilum* for humans and/or domestic animals is yet unclear.

*Theileria* (*B*.) *microti* was only detected in ticks from a peri‐urban location (Germia), a forest on the outskirts of Prishtina that is used for hiking and dog walking. Generally, the highest number of different pathogens (seven) was found there. Wild rodents might be reservoirs for *Th*. (*B*.) *microti* in the sampling area. Despite the absence at all other sampled locations, the detection of *Th*. (*B*.) *microti* may be of medical relevance as the main causative agent of human babesiosis in Europe (Hildebrandt et al. 2021). Few autochthonous cases been reported from European countries, and the pathogenicity of different strains to humans is still unclear. The first confirmed autochthonous case was identified from an immunocompromised patient living in Germany and caused by the “Jena strain,” closely related to the US genotype (Hildebrandt et al. 2007). A second strain “Munich,” originally presumed non‐zoonotic, is widely present in Europe, and recently DNA has been detected in immunocompetent patients with various symptoms following tick bites, which indicates infectivity of this strain to humans with potentially less pathogenicity (Moniuszko‐Malinowska et al. 2016; Siński et al. 2006). Because of its rarity, the awareness of babesiosis as an infection in humans is low and thus it is likely underdiagnosed. Here, we would also like to emphasise that *Th*. (*B*.) *microti* is an organism with still unclear taxonomy as it does neither truly belong to the *Babesia* nor to the *Theileria* genus based on molecular (18S rRNA) analyses (Uilenberg 2006).

We did not detect *F. tularensis* DNA or Flavivirus RNA in the sampled ticks. Several outbreaks of tularemia have been recorded in the last two decades in Kosovo. However, transmission involves several modes such as handling of infected animals, consumption of contaminated food or water, or vector bites (Yeni et al. 2020). Epidemiological studies have suggested that disrupted agricultural environments, deserted homes and unprotected food stores in post‐war Kosovo caused a rapid increase in rodent populations favourable for epizootic spread of tularemia in rodents and consequent widespread environmental contamination with *F. tularensis* (Reintjes 2002; Sadiku et al. 2013). Ticks likely played a minor role during those outbreaks and considering the low sample size of our study as well as no recent tularemia outbreaks, the absence of *F. tularensis* in our samples is not surprising.

Similarly, the status of TBE is unclear in Kosovo and its neighbouring countries. Venturi et al. (2011) did not observe anti‐TBEV antibodies in blood of 200 donors originating from the Peja region (locations #1 and #2 in our study are located in this region) and none of 340 *I. ricinus* specimens collected in different regions of Kosovo in 2014 and 2015 were positive for TBEV RNA (Sherifi et al. 2018). Considering that TBEV transmission cycles are very focal, even in highly endemic regions such as Austria (Heinz et al. 2015), the clarification of its current status in Kosovo would need a combined effort of surveillance of ticks and serosurveys.

We are aware that our study only covers a few regions in Kosovo and sampling activity is based on single surveys in April at the sampled locations. The sole detection of *I. ricinus* might be a result of the chosen time of the season, which could favour higher abundance of *I. ricinus* compared to other species. Sherifi et al. (2018) trapped three tick species by flagging, most abundantly *I. ricinus* , followed by *D. marginatus* , and *Haemaphysalis* spp., however, sampling efforts stretched over 2 years and two timepoints (April to June and September to October). Thus, further studies on the distribution of *I. ricinus* in other parts of Kosovo as well as on its seasonality should be conducted to further elucidate circulating pathogens and potential transmission cycles.

## 5 Conclusion

Our study clearly shows that tick‐borne pathogens of medical and veterinary relevance are present in *I. ricinus* ticks in Kosovo. Our data underline that particularly urban areas may serve as sources of infections for humans and animals as shown in other European countries (Corrain et al. 2012; Hansford et al. 2022; Sormunen et al. 2020). Because of limited available data, broader surveys including vector surveillance and serological surveys are necessary to assess species and pathogen diversity in Kosovo.

## Author Contributions

I.H., J.W., K.S. and E.K. designed the study. B.X., G.H., B.J., K.S. and E.K. conducted field work. I.H., M.W. and M.R. performed laboratory work. I.H., M.W., D.Ç., A.M., and E.K. analysed the data. I.H., M.W., A.G.O., M.W., J.W., K.S. and E.K. wrote the manuscript. Funding acquisition A.G.O., M.W. and J.W., K.S. and E.K. All authors read and approved the final manuscript.

## Ethics Statement

Data analysis only involved questing ticks sampled from the environment, neither human nor lab or any other vertebrate animal samples were analysed in this study, and thus no ethics approval was required.

## Consent

The authors have nothing to report.

## Conflicts of Interest

The authors declare no conflicts of interest.

## Data Availability

All data generated and analysed during this study were included in the article.
